# Postoperative lymphatic recurrence distribution and delineation of the radiation field in lower thoracic squamous cell esophageal carcinomas: a real-world study

**DOI:** 10.1186/s13014-022-01987-7

**Published:** 2022-03-05

**Authors:** Rongxu Du, Songqing Fan, Xiaobin Wang, Xia Hou, Cheng Zeng, Dan Guo, Rongrong Tian, Dan Yang, Leilei Jiang, Xin Dong, Rong Yu, Huiming Yu, Dongming Li, Shuchai Zhu, Jie Li, Anhui Shi

**Affiliations:** 1 Key Laboratory of Carcinogenesis and Translational Research (Ministry of Education/Beijing), Department of Radiation Oncology, Peking University Cancer Hospital and Institute, Beijing, 100142 People’s Republic of China; 2 Oncology Division I, China Pingmei Shenma Medical Group General Hospital, Kuanggongzhong Rd.1, Xinhua District, Pingdingshan, 450052 Henan People’s Republic of China; 3 Department of Radiation Oncology, The Fourth Hospital of Hebei Medical University and Hebei Cancer Hospital, JianKang Rd.12, Shijiazhuang, 050011 Hebei People’s Republic of China; 4 Department of Radiation Oncology, Shanxi Cancer Hospital, No.3 Workers New Village, Xinghualing District, Taiyuan, 030013 Shanxi People’s Republic of China; 5 Department of Radiation Oncology, Central Theater General Hospital, Wuluo Rd.627, Wuchang District, Wuhan, 430061 Hubei People’s Republic of China

**Keywords:** Real-world study, Lower thoracic esophageal carcinomas, Postoperative lymph node metastasis, Clinical target volume delineation

## Abstract

**Background:**

To study lymphatic recurrence distribution after radical surgery in the real world and guide clinical tumor volume delineation for regional lymph nodes during postoperative radiotherapy for lower thoracic squamous cell esophageal carcinomas.

**Methods:**

We enrolled patients who underwent radical esophagectomy, without radiation before or after surgery, at 3 cancer hospitals. Patients were classified into groups according to tumor locations. We included patients with tumors in the lower thoracic segment and analyzed the postoperative lymph node recurrence mode. A cutoff value of 10% was used to differentiate high-risk lymph node drainage areas from others.

**Results:**

We enrolled 1905 patients in the whole study series, including 652 thoracic esophageal carcinomas that met our inclusion criteria; there were 241 cases of lower thoracic esophageal carcinomas. 1st, 2nd, 4th, 7th, 8th groups of lymph nodes, according to the 8th edition of the AJCC classification, displayed as high-risk recurrence areas, representing 17.8%, 23.9%, 11.7%, 10.9% and 12.2% of lymph node recurrence. Stage III-IV tumors located in the lower segment of the thoracic esophagus showed a tendency to recur in the left gastric nodes (7.9%) and celiac nodes (10.6%).

**Conclusions:**

According to our results, we recommended including the 4th, 7th and 8th groups of lymph nodes in the radiation field, and for patients with stage III-IV disease, the 17th and 20th groups of nodes should be irradiated during postoperative treatment. Whether including 1st/2nd groups in preventive irradiation needed more proofs.

## Background

Esophageal carcinoma is one of the most common malignant tumors in the world, ranking as the 4th leading cause of cancer-related death [1]. In Asia, squamous cell carcinoma accounts for the vast majority of esophageal carcinomas [2]. In China, the diagnoses and deaths in China accounted for 50% of all esophageal carcinoma patients worldwide in 2015 [3], and 90% of cases were squamous cell carcinomas [4]. At present, the major therapies for esophageal squamous cell carcinoma are comprehensive treatment based on surgery combined with chemotherapy and radiotherapy. The rate of surgical resection in China can reach 90–97% [5], and the surgical method has always been esophagectomy combined with two-field or three-field lymph node dissection. The locoregional recurrence rate after esophagectomy has been reported to be 17.0–41% in previous studies [6, 7].

Since many patients in China tend to choose surgery as the first treatment, adjuvant radiotherapy could play a more important role in the treatment of Chinese esophageal carcinoma patients. Adjuvant radiotherapy has been suggested to be beneficial for decreasing the recurrence of esophageal carcinomas and increasing overall survival, especially in patients with stage II–III disease or with positive lymph nodes [8–13]. The current National Comprehensive Cancer Network (NCCN) guidelines recommend surveillance over adjuvant treatment in patients after preoperative chemoradiation and surgery [14], as preoperative chemoradiotherapy has become a standard of care in the United States during the last decade [15] and has brought a pathological complete response rate of 20–30% [16]. A National Cancer Database Analysis reported an increase in the percentage of patients undergoing neoadjuvant treatment followed by surgery, from 29% in 2004 to 40% in 2014 [17]. However, the application of neoadjuvant treatment in Chinese esophageal carcinoma patients is not that widespread. According to a study guided by the Esophageal Cancer Committee of the China Anti Cancer Association, the rates of neoadjuvant radiotherapy, neoadjuvant chemotherapy, postoperative radiotherapy and postoperative chemotherapy reached 2.0%, 2.0%, 7.0% and 26.0%, respectively, in approximately 2012 [18]. As a result, Chinese experts still recommend taking more active steps for patients without preoperative treatment to promote local control and prevent recurrence. However, the specific clinical tumor volume (CTV), especially the clinical tumor volume of regional nodes (CTVn), remains controversial, as the metastatic lymph nodes identified by surgical pathologic results are not necessarily the nodes that have a high risk of recurrence after surgery, and the tumors located in different segments tend to have different recurrence modes. Thus, it is important to clarify the lymph node areas that should be included in treatment to achieve more precise radiotherapy.

## Methods

From January 1st, 2014, to December 31st, 2019, 1905 patients with thoracic esophageal cancers underwent radical esophagectomy at the Department of Thoracic Surgical Oncology at 3 clinical centers, namely Beijing Cancer Hospital, Hebei Cancer Hospital and Shanxi Cancer Hospital, located in central areas, which was typical of the high incidence of esophageal carcinomas in China [19]. According to the eighth edition of the American Joint Committee on Cancer (AJCC) criteria, the location of the primary tumor was defined by the center of the tumor, with endoscopic measurements of each region measured from the incisor. Thoracic esophageal carcinoma was defined as a primary tumor located in the esophageal segment measured from the incisor, ranging from 20 to 40 cm, while the lower thoracic segment was defined as the part of the esophagus from the inferior pulmonary vein to the lower esophageal sphincter (30–40 cm away from the incisors) [20]. The lymph node groups included in our study were also defined by the 8th edition of the AJCC criteria [20].

In this retrospective study, the inclusion criteria for enrollment were as follows: 1) patients aged between 18 and 80 years old; 2) the tumor was confirmed to be located on the lower segment of the thoracic esophagus by endoscopy, esophagography, computed tomography (CT), or positron emission tomography computed tomography (PET-CT) before esophagectomy; 3) the tumor was confirmed by postoperative pathology to be clearly diagnosed as squamous cell esophageal carcinoma; 4) patients were pathologically confirmed to have R0 resection; 5) patients had sufficient clinical materials and imaging results that included all the treatment history and follow-up; and 6) lymphatic recurrence was confirmed by PET-CT or continuous enhanced CT scan. We considered the lymph nodes as positive when they met the following criteria: ① the short axis was greater than 1 cm, as shown by enhanced CT images, while the tracheoesophageal groove lymph nodes had a short axis greater than 0.5 cm, or the suspicious lymph nodes became gradually enlarged during the observation period; ② the lymph nodes had clear, high uptake of FDG in PET-CT (SUV ≥ 2.5); ③ the patients that failed to meet the conditions above but were highly suspected to have recurrence were confirmed by biopsy pathological diagnosis or a multiple-disciplinary team (MDT).

The exclusion criteria were as follows: 1) patients histologically diagnosed with adenocarcinomas or other nonsquamous cell histological types; 2) patients with cervical esophageal carcinomas or upper/middle thoracic esophageal carcinomas; 3) patients who received neoadjuvant or adjuvant radiotherapy before the confirmation of recurrence; 4) patients with more than one primary tumor; and 5) patients lacking important clinical information, e.g., pathological results and surgical records. In addition, since we acquired the clinical data from retrospective perspective and didn’t intervene any treatment of patients, there was no ethics approval involved in our study.

We used SPSS 24.0 (SPSS Inc., Chicago, IL, USA) to organize data and perform chi-square tests to determine the factors related to lymph node recurrence.

## Results

During a median follow-up period of 41 months (6–76 months), there were 652 patients met the criteria, and we analyzed 241 cases of lower thoracic esophageal carcinoma in this study. As shown in Table 1, the patients age ranged from 40 to 80 years old, with a median age of 59. Male patients accounted for 86.7% of the whole group, while female patients made up 13.3%. Pathologic diagnosis showed 47 patients had stage 0-I disease, 71 patients had stage II disease, and 123 patients had stage III-IV disease. There were 119 patients with highly or moderately differentiated tumors and 47 patients with poorly differentiated tumors, and the grade of differentiation of 75 patients was unclear. In regard to the surgical methods, 63 patients underwent two-incision surgery (right thoracotomy and midline laparotomy), and 71 underwent three-incision surgery (right thoracotomy, midline laparotomy and left cervical incisions). 29.0% patients accepted neoadjuvant or adjuvant chemotherapy. There were 140 patients with recurrence in the lymph nodes after therapy and 247 recurrent lymph node loci in total (Table 2).

**Table 1:** The clinical characteristics of the patients enrolled in the study

Characteristics	Patients	
	No	Constituent ratio (%)
Age	40–80 y (59 y)	
Sex		
Male	209	86.7
Female	32	13.3
Pathological type		
Squamous cell carcinomas	241	100
Stage (AJCC 8th)		
Stage 0	11	4.6
Stage I	36	14.9
Stage II	71	29.5
Stage III	93	38.6
Stage IV	30	12.4
Grade of differentiation		
Highly differentiated	10	4.2
Moderately differentiated	109	45.2
Poorly differentiated	47	19.5
Undefined grade	75	31.1
Location		
Lower thoracic	241	100
Chemotherapy		
Neoadjuvant + /adjuvant chemotherapy	70	29.0
Without chemotherapy	171	71.0
Surgical method		
Two-incision surgery	63	26.1
Three-incision surgery	71	29.5
Other methods or undefined methods	107	44.4
Recurrence		
LN recurrence	140	58.1
No LN recurrence	101	41.9
Total	241	100

**Table 2:** The distribution and rate of lymph node recurrence in lower thoracic esophageal carcinoma

Lymphatic drainage area	Recurrence number in nodes	Percentage
1	44	17.8
2	59	23.9
4	29	11.7
7	27	10.9
8U	1	0.4
8M	15	6.1
8Lo	14	5.7
9	0	0.0
16	13	5.3
17	17	6.9
18	6	2.4
19	0	0.0
20	22	8.9
Upper cervical	0	0.0
Total	247	100.0

The lymphatic recurrence rate was 58.1% in our study. Our results also showed that lower thoracic esophageal carcinoma was characterized by recurrence in the thoracic paraesophageal nodes (12.2%), which was more concentrated in the middle and lower groups, and abdominal nodes, especially in the left gastric nodes (6.9%) and celiac nodes (8.9%). It also tended to have lymph node recurrence in the subcarinal nodes (10.9%) and lower paratracheal nodes (11.7%). Notably, the 1st and 2nd groups of lymph nodes accounted for 17.8% and 23.9% of the lymph node recurrence of lower esophageal carcinomas, respectively.

We set the cut-off value at 10% to differentiate high-risk lymph node drainage areas that need to be included in treatment to avoid recurrence; that is, we recommended irradiating the regions where the lymph node recurrence rate was higher than 10% in our target radiation field. According to this threshold value, we could identify high-risk areas based on our statistical analyses.

The 4th, 7th and 8th groups of lymph nodes were shown to be at a high risk of recurrence in lower thoracic esophageal cancer. In addition, it should be noted that the left gastric nodes and celiac nodes also showed a relatively high rate. However, our results also showed that station 1–2 lymph nodes should be included in prevention areas, which needs further analysis. As shown in Table 3, we compared the recurrence in lymph node groups including the 1st and/or 2nd node group, and in other lymph node groups excluding these two groups, we found that 17.1% of all the recurrent lower thoracic esophageal carcinoma patients had recurrences in 3 or more lymph node groups, patients with multiple lymphatic recurrence sites (≥ 3) always tended to include the 1st and 2nd groups in the recurrence patterns (*P* = 0.007), to be exact, 83.3% of patients with 3 or more than 3 groups of positive lymph node had recurrence in these two groups. There was a significant difference between the patients with stage (y)pT0-T2 and stage (y)pT3-T4 for lymphatic recurrence in the 1st and 2nd groups (*P* = 0.025). Additionally, those with stage (y)pT0-T2 disease seemed to have a higher recurrence rate in stations including 1st and/or 2nd than the others, as 69.4% of (y)pT0-T2 patients had recurrences in the 1st and/or 2nd groups of nodes. There was no significant difference in the patients with different AJCC stages (*P* = 0.112), different surgical methods (*P* = 0.235), with or without anastomotic recurrence (*P* = 0.199), and patients with or without concurrent distant metastasis (*P* = 0.500).

**Table 3:** Factors related to recurrence in the 1st and 2nd lymph node groups

Variables	Recurrence in lymph node groups including the 1st and/or 2nd group	Recurrence in other lymph node groups except for the 1st or 2nd group	*P* value
Invasion depth			
(y)pT0-T2	43	19	0.025
(y)pT3-T4	42	36	
AJCC stage			
0–II	37	27	0.112
III–IV	48	28	
Anastomosis condition			
Anastomotic recurrence	5	5	0.199
Without defined anastomotic recurrence	80	50	
Concurrent distant metastasis			0.500
Yes	19	8	
No	66	47	
Number of metastatic LN groups			
≥ 3 recurrent lymph node groups	20	4	0.007
< 3 recurrent lymph node groups	65	51	
Surgical method			
Two-incision surgery	25	13	0.235
Three-incision surgery	25	20	

**Fig. 1:**
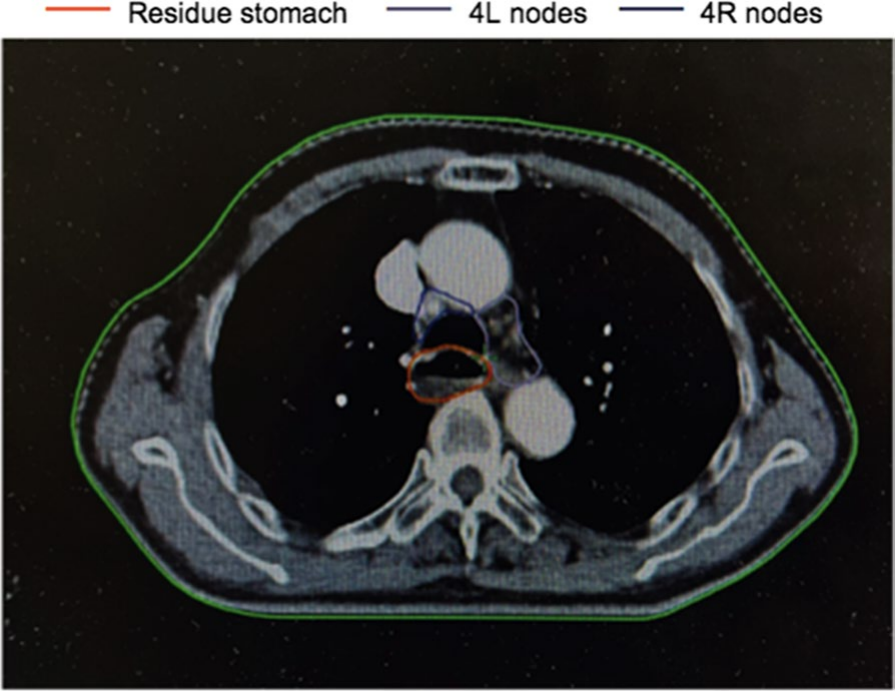
The area of 4th lymph node group in target delineation

**Fig. 2:**
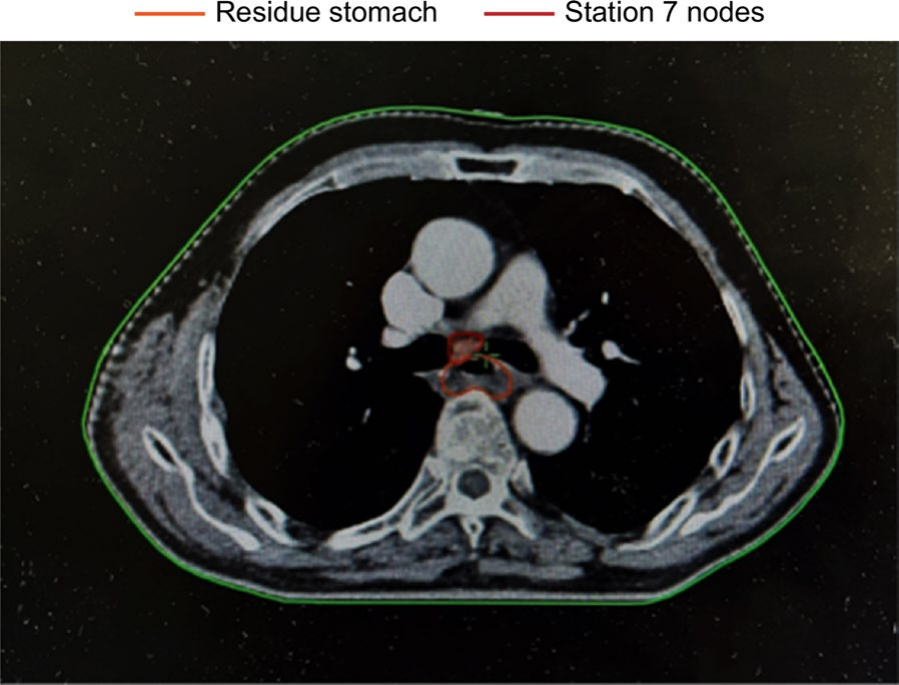
The area of 7th lymph node group in target delineation

**Fig. 3:**
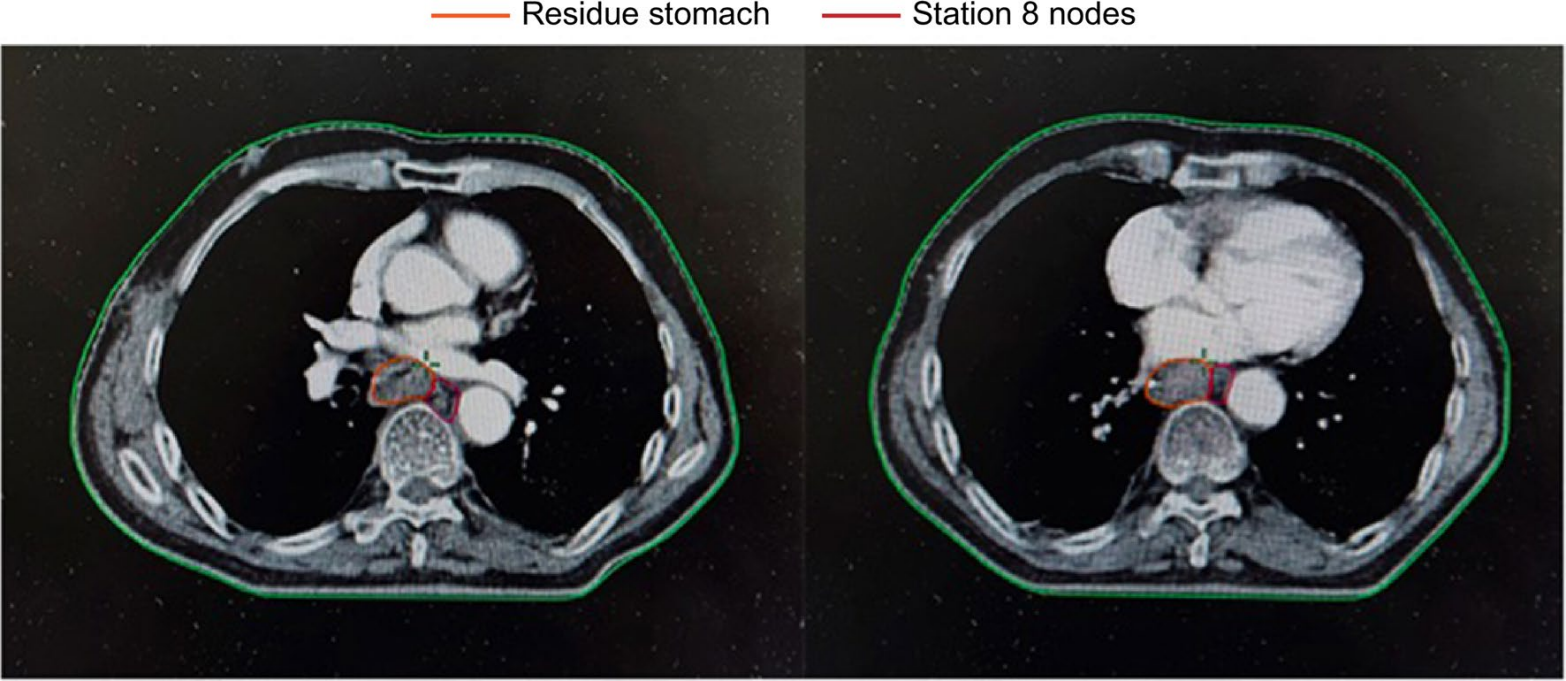
The area of 8th lymph node group in target delineation

We showed the delineation of the lymph node areas involved in our radiation field in Figs. 1, 2 and 3, through a realistic case of lower thoracic esophageal cancer, including the delineation of 4/7/8 lymph node areas. The definition of the border was based on the IASLC lymph node map [21].

## Discussion

Surgery is a major radical treatment for esophageal carcinomas, especially for middle and lower thoracic esophageal cancers. However, since there are massive lymphatic vessels, vessels and important organs adjacent to the esophagus, it is difficult to perform complete primary tumor resection and lymphadenectomy, thus, subclinical lesions or residual tumor can remain and result in regional recurrence or distant metastasis. Due to the lymphatic drainage characteristics of the esophagus, lymphatic recurrence remained at 24–40% even after high-quality surgery, and the overall recurrence of lymph nodes after esophagectomy combined with three-field lymphadenectomy still reached 40–57.8% [22–26], similar to our results. The recurrence rate of 58.1% shown in our study seems to be slightly higher than that in most previous studies, but it might result from that 51.0% of the patients in our study were stage III-IV, which might suggest a higher possibility of recurrence, since deeper invasion of the primary tumor always means higher recurrence rates [27]. Additionally, the surgery quality could have been different, and it has been difficult to meet a common standard in different clinical centers. Katayama et al. [28] also reported that despite sweeping the lymph nodes, the rate of recurrence could still be suboptimal. The high incidence of early recurrence and poor long-term prognosis of patients are the reasons that more specialists recommend postoperative treatment in China [29].

Previous studies in China have considered adjuvant radiotherapy as a necessary treatment to prevent local recurrence and improve the prognosis of patients, and this has been especially preferred in patients with stage III-IV disease or with positive nodes [10, 30–32]. In regard to the specific radiation field of radiotherapy, bilateral supraclavicular area, the whole mediastinum and part of pericardia and left gastric regions have been recommended for inclusion in the irradiation field to cover as much of the lymphatic region as possible. However, this field could be too large to bring about unnecessary side effects of radiotherapy, affecting the prognosis and quality of life, which might not be offset by the decrease in recurrence.

The delineation consensus [33] suggested that the border of the clinical tumor volume was 3–4 cm away from the proximal terminal of the gross tumor and 4 cm away from the distant terminal of the lower thoracic esophageal tumor, including all suspected lymph nodes with an extension distance of 1 cm around the nodes. However, with the development of radiation technology, the radiation field can be more precisely controlled to decrease radiation injuries; thus, the patterns of lymphatic drainage around the esophagus could be key to designing the radiation field. As a result, there have been more studies discussing more precise delineation according to the different rates of recurrence in each lymphatic drainage area, including our study.

Generally, the lymph nodes around the esophagus drain into three areas, the lower cervical lymph node area, thoracic mediastinum and abdominal cavity, depending on the location of the tumor [34]. Lower thoracic esophageal cancer predominantly metastasizes downward, with a higher rate of recurrence in stations 16–20 than tumors in other locations [35]. Accordingly, prophylactic irradiation of the upper abdominal lymph nodes should be considered, whereas this treatment is not indispensable for upper and middle thoracic esophageal cancers [36]. Our results also indicated a high rate of lymph node recurrence in abdominal nodes, in accordance with the recurrence trends reported by previous studies, but the recurrence rate of stations 16, 17 and 20 failed to reach our threshold value of 10%. To clarify the necessity of including these groups of nodes in the radiation field, we further analyzed the recurrence rate in a subgroup of stage III-IV patients. We observed 123 patients with stage III-IV disease and 151 lymph node (LN) recurrence sites, among which 12 positive nodes were found in station 17, and 16 positive nodes were located in station 20; the proportions were 7.9% and 10.6%, respectively. These rates were higher than the recurrence incidence of the whole group of patients, suggesting that stronger recommendations should be made to include celiac lymph nodes in lower thoracic esophageal carcinoma patients with stage III–IV disease.

In addition, there were some discrepancies between our results and those of previous studies. Previous retrospective studies showed a relatively low rate of lymph node recurrence in the supraclavicular area or upper mediastinal area, mostly lower than 5% [37], which seemed to contradict our results. Thus, we tried to find the explanation through analyzing possible factors affecting recurrence in different lymph node groups, the results showed that patients with multiple lymphatic recurrence sites (≥ 3) tended to include the 1st and 2nd groups in the recurrence patterns, and patients with stage (y)pT3-T4 disease tended to have recurrence in multiple lymph node groups. Additionally, those with stage (y)pT0-T2 disease seemed to have a higher recurrence rate in stations 1–2 than the others. Although advanced tumors are more likely to have lymph node recurrence, due to the special structure of the thoracic esophagus, the recurrent lymph nodes of early-stage patients (pT1b-T2) are likely to skip to the boundary between the lower cervical and upper thoracic areas, and the rate of paraesophageal node recurrence rapidly increases only after primary tumors invade the adventitious coat of the esophagus [38]. Wang et al. [39] also indicated that the complex structure of large blood vessels and nerves surrounding the esophagus, especially near the recurrent laryngeal nerve, greatly increases the difficulty of sweep and may lead to the omission of latent positive lymph nodes. These findings may explain why (y)pT0-T2 patients in our study were more likely to have recurrence in the lower cervical paraesophageal and upper paraesophageal areas.

Doki et al. [40] reported that in squamous cell esophageal carcinoma patients who underwent radical surgery without preoperative treatment, recurrence most frequently occurred in the cervical nodes (19%), abdominal para-aortic nodes (17%), and upper mediastinal nodes (17%). Therefore, some studies also suggested that stations 1–5 and 7 should be included in the irradiation field for the postoperative treatment of thoracic esophageal cancers regardless of the location of the primary tumors, and the upper abdominal lymph nodes could be high-risk nodes for lower cancers [36, 41]. As locoregional lymph node recurrence has been a major type of postoperative failure in squamous cell esophageal carcinoma [36, 42], preventing lymphatic recurrence is definitely important in multimodal therapy, and more studies determined that the lymph node groups in the whole mediastinum and upper abdominal area were at a high risk for the recurrence of lower thoracic cancer [39, 43, 44].

In summary, postoperative radiotherapy has been suggested for patients with a high risk for local recurrence, such as pT3–T4 carcinomas, positive lymph nodes, and close/positive margins [45]. Yu et al. [1] showed that the upper and middle mediastinal regions were the most common sites of lymph node metastases for tumors in all segments of the thoracic esophagus and suggested that the upper abdominal region should be irradiated in patients with a pathological stage of IIIB or higher. Combined with our results, undoubtedly, the 4th, 7th, 8th groups of lymph nodes should be included in postoperative radiation, and it does not seem safe to exclude the 1st and 2nd groups even in lower cancers, especially in patients with a tendency to have multiple-station metastases, patients with early T stages may be also more likely to have recurrence in the 1st and 2nd lymph node groups. But whether including the 1/2 node groups in regular recommendation of irradiation for all the patients need more confirmation, in view of the toxicities after wide range of radiation. In patients with advanced-stage disease and other high-risk factors, the upper abdominal region should be considered for coverage in the treatment field.

There were some limitations in our study. First, it was difficult to standardize the standard treatment and surgery quality at each clinical center, which could have affected the recurrence and prognosis of patients. Second, we collected the clinical data from medical records, and while we concentrated on the recurrence of lymph nodes, there may have been missing records, such as the survival and pre-operative condition of patients, and the related analysis could have been suboptimal. Finally, more evidence is needed to address whether it is safe to exclude the 1st and 2nd groups during delineation for lower thoracic tumors.

## Conclusions

In conclusion, we recommend that at least the 4th, 7th and 8th groups of lymph nodes be covered in the radiation field to prevent postoperative recurrence. Additionally, for patients with high-risk factors, such as advanced stage, the 17th and 20th groups of nodes should be considered for irradiation in postoperative treatment. Further study is needed to determine whether to irradiate the 1st or 2nd group of nodes in lower thoracic esophageal carcinomas.

## Data Availability

All data generated or analyzed during this study are included in this published article.

## Abbreviations

AJCC: American Joint Committee on Cancer
NCCN: National Committee on Computer Network
CTV: Clinical tumor volume
CTVn: Clinical tumor volume of regional nodes
MDT: Multiple-disciplinary team
CT: Computed tomography
PET-CT: Positron emission tomography computed tomography
